# Strategic Applications of Single-Atom Skeletal Editing in Natural Product Synthesis

**DOI:** 10.1021/jacs.6c04423

**Published:** 2026-05-22

**Authors:** Reem Al-Ahmad, Isabel M. de la Torre Roehl, Chang Liu, Richmond Sarpong, Mingji Dai

**Affiliations:** † Department of Chemistry, 1371 Emory University, Atlanta, Georgia 30322, United States; ‡ Department of Chemistry, 1438 University of California, Berkeley, California 94720, United States; § Department of Pharmacology and Chemical Biology, School of Medicine, Emory University, Atlanta, Georgia 30322, United States

## Abstract

Skeletal editing is rapidly reshaping synthetic chemistry by enabling precise, atom-level modifications to complex molecular frameworks. Despite recent advances in methodology development, skeletal editing has yet to realize its full impact on complex molecule synthesis. In this Perspective, we highlight the power of single-atom skeletal editing transformations in enabling efficient syntheses of various complex natural products and advocate for the development of new methods and novel applications. We present guidelines rooted in historical precedent along with modern case studies that highlight when skeletal edits enable efficient synthesis, and where reactivity gaps still limit broader adoption. By analyzing the existing literature, we aim to inspire novel retrosynthetic disconnections, and steer new method development, which, collectively, will advance the fields of total synthesis and skeletal editing.

## Graphical abstract

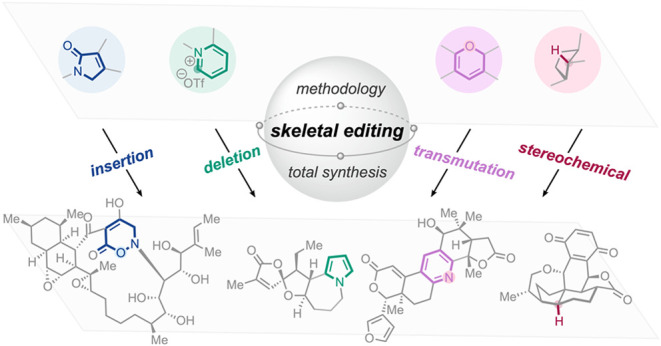

Skeletal editing has gained momentum in recent years, evolving from a conceptual aspiration into one of the most rapidly growing areas for method development in modern synthetic chemistry. Synthetic tools for skeletal editing enable chemists to reconfigure ring size or atom identity—through insertions, deletions, or transmutations—without changing the rest of the molecule. The ability to precisely edit individual atoms within a molecular skeleton is changing the way chemists build molecules. Many emerging skeletal editing transformations have been covered in recent reviews,^1–6^ however, understanding their utility and limitations in the context of total synthesis remains essential to guide future directions in method development. While both single- and multiatom skeletal edits serve as powerful tools for manipulating molecules, in this Perspective, we will focus on the application of single-atom skeletal editing strategies in natural product synthesis.

For more than a century, complex natural product synthesis has evolved alongside the development of synthetic methods. Because of their structural diversity and complexity, natural products often inspire new methods, but also reveal both the capabilities and limitations of existing methods.^7^ This iterative feedback loop, where practice informs method and method redirects practice, has driven many advances in organic synthesis. An early example of this iterative process is the discovery of the Nobel Prize winning Diels-Alder reaction in 1928. The Diels-Alder cycloaddition was immediately recognized for its potential to construct complex polycyclic ring systems.^8,9^ Its first applications in the total synthesis of natural products came in the early 1950s with Woodward’s cholesterol synthesis,^10^ Merck’s cortisone synthesis,^11^ and Stork’s cantharidin synthesis.^12^ The need for Diels-Alder reactions that achieve high degrees of regioselectivity, stereoselectivity, and enantioselectivity led to the development of the Danishefsky-type synergistic dienes, Lewis acid catalyzed variants, as well as chiral auxiliary- or catalyst-controlled diastereo- or enantioselective versions. These new advancements enabled additional natural product total syntheses. This iterative feedback loop linking molecular targets, synthetic strategy, and reaction development is not limited to the Diels-Alder reaction and is a common pattern in the evolution of most impactful synthetic methodologies, including olefin metathesis,^13^ palladium-catalyzed cross couplings,^14^ organocatalysis,^15^ and C–H functionalization,^16^ among others.

This productive feedback loop is now fueling the field of single-atom skeletal editing. In many total synthesis endeavors, the atoms in molecular skeletons—the cyclic core framework—are often treated as fixed or static. Skeletal editing challenges this view by treating molecular skeletons as programmable frameworks, primed for atom-level edits en route to a target molecule. Here, we define single-atom skeletal editing as the modification of a given ring system (as opposed to peripheral substituents) by a singular atom. To maintain conceptual clarity, we provide a distinction between skeletal editing and structural remodeling. In our view, the latter refers to the changing of multiple ring systems within the same step and often proceeds via rearrangements. This distinction allows for a clear and consistent categorization of classical and modern skeletal transformations in this Perspective.

## Classification Framework for Single-Atom Skeletal Editing

In the context of complex molecule synthesis, we posit that skeletal editing amounts to more than a late-stage diversification strategy. In our opinion, it offers an encoded disconnection strategy that is embedded within the retrosynthetic plan from the outset. Skeletal edits vary widely in their impact on the synthetic strategy in a given total synthesis. As such, we have developed a decision-making framework to rigorously identify skeletal editing transformations that meaningfully reshape synthetic logic and may therefore find wider application in the future (Figure 1A). First, the skeletal editing step should be clearly defined and strategically enabling to the overall synthesis. Specifically, the skeletal edit should enhance synthetic efficiency and atom economy through a late-stage transformation on a previously installed, encoded, functionality. These edits often serve as points of divergence, enable access to challenging skeletons, yield key stereochemical outcomes, or allow for reactivity otherwise inaccessible on the targeted molecular scaffold. In certain cases, skeletal edits can act as temporary core modifications that facilitate key transformations en route to a target molecule. A prominent example is Corey’s synthesis of (±)-prostaglandin F2α (**3**), where a carbonyl-based oxygen-atom insertion (Baeyer-Villiger oxidation) enabled the cleavage of a transient cyclohexene moiety (**1**) formed via a key enantioselective Diels-Alder cycloaddition, which installed three of the four contiguous stereocenters present in the natural product (Figure 1B).^18^ While these kinds of strategic applications of skeletal edits can be extremely enabling in synthesis, to most clearly illustrate their utility, we have chosen to only highlight examples in which the ring system generated as a result of the edit is retained in the target molecule.

**Figure 1:**
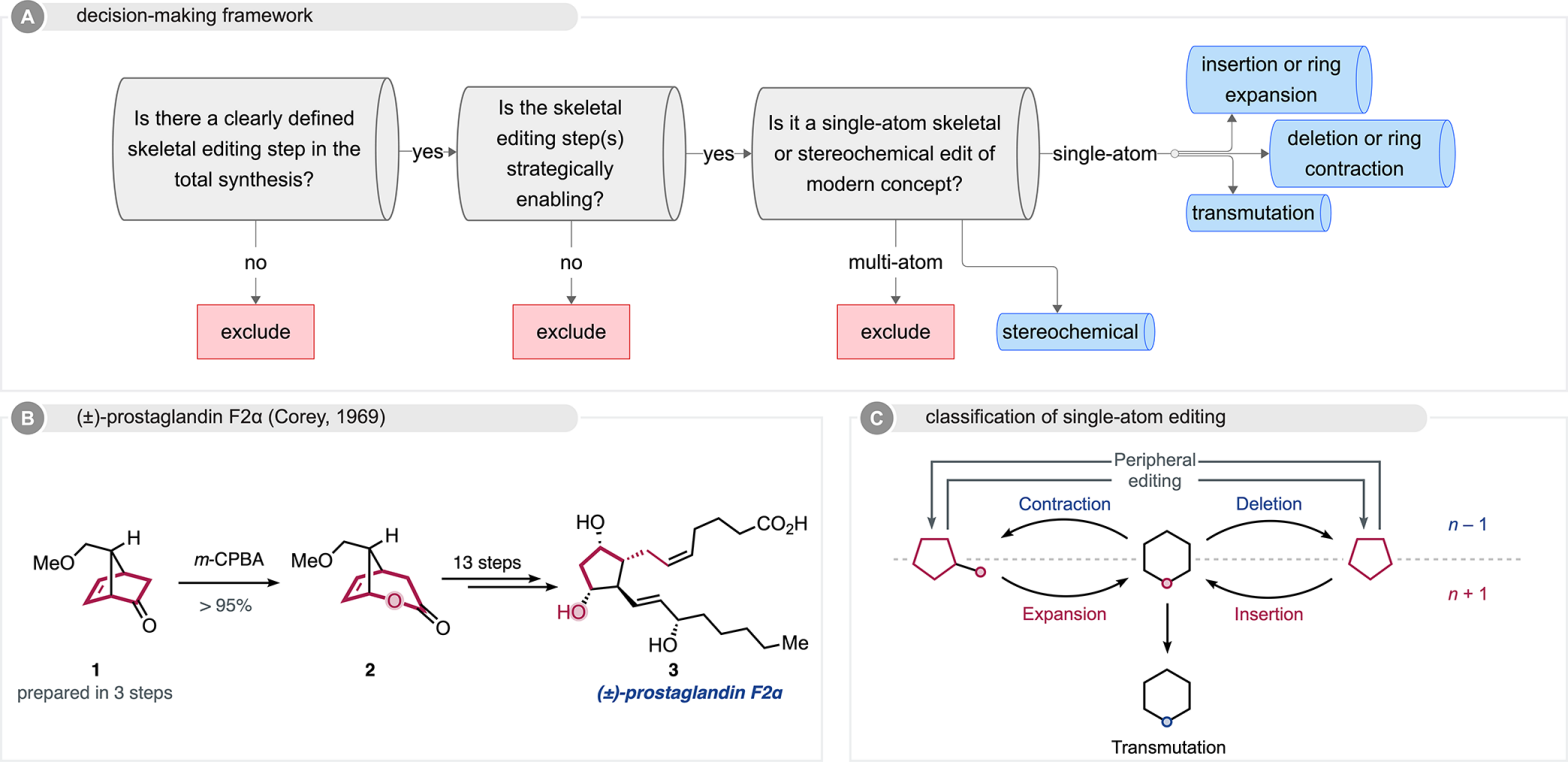
Single-atom skeletal editing classification. (a) Decision making framework. (b) (±)-Prostaglandin F2α by Corey. (c) Classification of single-atom editing (adapted with permission from Sarpong and Levin et al.^17^ Copyright 2022 Springer Nature Limited).

For clarity, we differentiate single-atom from multiatom skeletal edits. The latter type of skeletal editing is powerful but falls outside the scope of this Perspective. We include single-atom stereochemical editing, defined here as selective changes to the configuration of an atom within a skeleton that preserves atom count and connectivity but alters three-dimensional shape. Although stereochemical editing describes a modification that changes how a peripheral substituent is disposed, it is often strategically necessary in order to access biologically relevant scaffolds or correct a stereocenter that resides on a core framework. We will highlight cases where challenging epimerizations were enabled by modern and innovative stereochemical editing techniques.

Syntheses are discussed in the context of the category of skeletal edit applied—insertion or ring expansion, deletion or ring contraction, transmutation, and stereochemical editing (Figure 1C). As established by Sarpong and Levin et al., the classification of skeletal edits depends on whether the core ring system (an *n*-membered ring) has expanded (*n* → *n* + 1) or contracted (*n* → *n* – 1).^17^ If the atom being edited resides on the starting material or is retained in the product as an exocyclic substituent of the ring system, we refer to the transformations as ring expansions or contractions, respectively. In contrast, insertions and deletions describe transformations in which the atom being edited is not directly attached to the ring system in question. If the core ring system has undergone an exchange of a constituent atom (*A* → *B*; no net change in ring size), we refer to this type of transformation as a single-atom transmutation as previously defined.^17^

Although the term skeletal editing has gained prominence in recent years, its underlying logic is deeply rooted in classical total synthesis. Carbonyl-based transformations such as the Tiffeneau-Demjanov rearrangement, Baeyer-Villiger oxidation, and Favorskii or Wolff rearrangements are well-established, elegant methods that accomplish single-atom skeletal modification. Though these reactions have not been framed as “skeletal edits”, they achieve many of the same goals with remarkable foresight. What we now recognize as skeletal editing strategies are built upon a foundation laid by many pioneers. For these well-established and classical modes of skeletal modification, we have chosen to highlight only representative examples as their application to total synthesis has been well documented.^19–30^ These methods are complementary to modern methods for single-atom skeletal editing, which look to engage other functional groups, beyond carbonyls, for skeletal modification.

## Insertions and Ring Expansions

Single-atom insertions and ring expansions offer a unique opportunity to introduce plug-and-play complexity into a scaffold with surgical precision. These transformations enable the conversion of commercially available or readily synthesized ring systems into larger (*n* + 1) ring systems embedded in the natural product cores, which are otherwise difficult to construct. The insertion of oxygen, nitrogen, or carbon atoms into ring systems represents a powerful late-stage strategy, particularly given the ubiquity of the resulting carbocycles and heterocycles in complex natural products.

### Oxygen-Atom Insertion

Given the prevalence of oxygen atoms in biologically active small molecules, especially natural products, oxygen-atom insertion comprises a strategically important skeletal editing subset, widely applied in organic synthesis to enable scaffold modification and diversification.^31–34^ As one of the earliest classical reactions, the Baeyer-Villiger oxidation enables the insertion of an oxygen atom adjacent to a carbonyl group in one step, expanding cyclic ketones into lactones. While classical Baeyer-Villiger conditions require a peracid,^35^ transition metal-catalyzed Baeyer-Villiger reactions have enabled a reversal of regioselectivity^36^ and enantioselectivity control.^37^ One classical example of this reactivity is found in the total synthesis of (±)-ginkgolide B (**6**) by Corey et al. (Scheme 1A).^38^ In this work, the 5/4/5/5-tetracyclic precursor (**4**) was efficiently accessed using a ketene-olefin [2 + 2] cycloaddition to install three contiguous stereocenters including one quaternary center. This cyclobutanone was primed for a subsequent Baeyer-Villiger oxidation which proceeded with high regioselectivity, revealing the target lactone embedded in the ginkgolide framework.

**Scheme 1:**
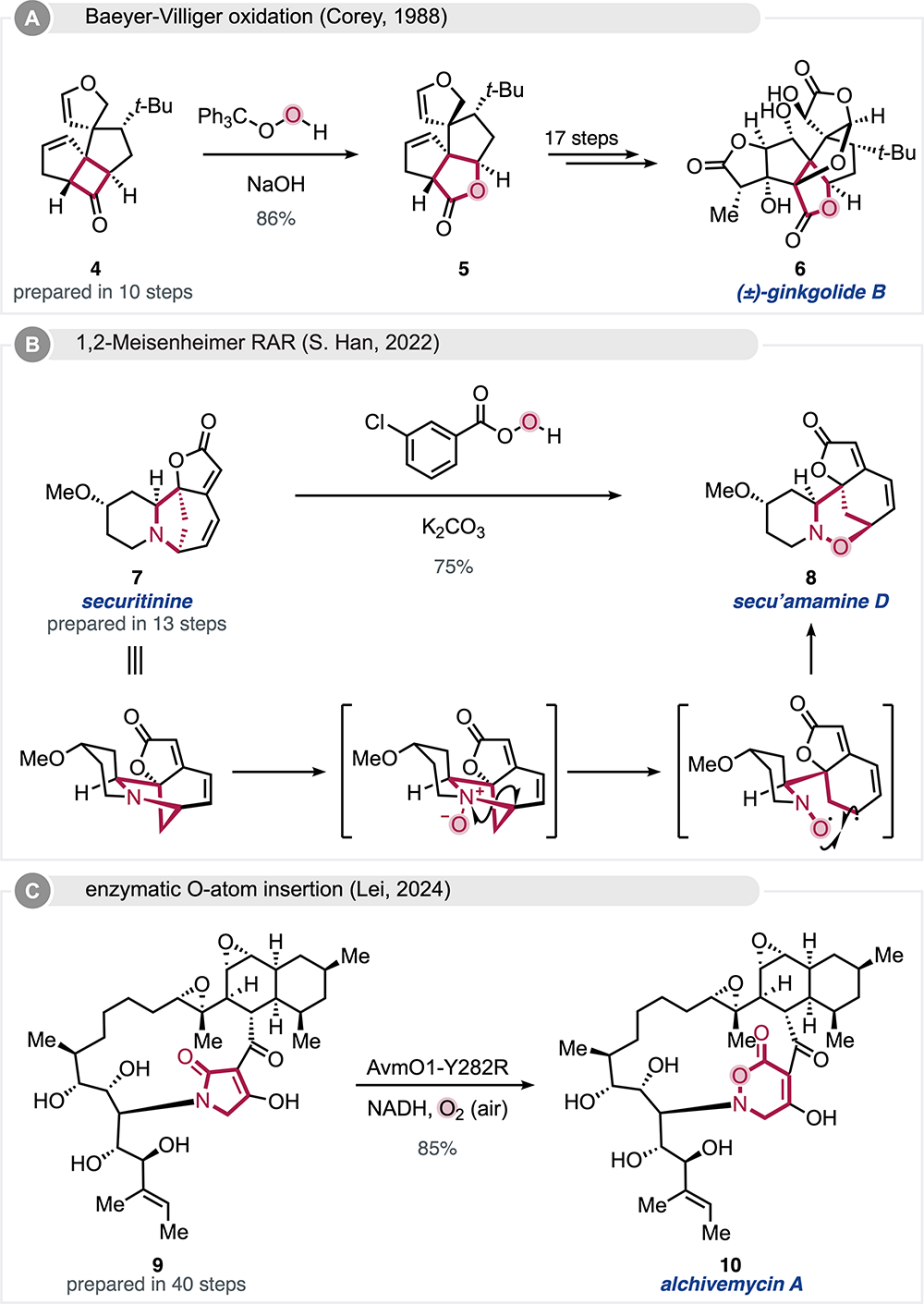
Representative Examples of Oxygen-Atom Insertion

A recent and unique example of oxygen-atom insertion was showcased in the 2022 total synthesis of C4-oxygenated securinine-type alkaloids by S. Han and co-workers (Scheme 1B).^39^ Deviating from a reliance on the classical carbonyl system, in this work an oxygen atom was inserted into a saturated C–N bond in one step, realizing a complementary dimension of reactivity. This oxidative rearrangement was first reported on securitinine (**7**) by Chen et al. in 2012,^40^ and later served as the inspiration for Ye and Wang’s 2018 biosynthetic proposal for securinine alkaloids.^41^ Mechanistically, it is proposed that treatment of (**7**) with *m*-CPBA and potassium carbonate leads to the oxidation of the tertiary amine group, which then undergoes a 1,2-Meisenheimer radical anion rearrangement (RAR) through β-scission and radical recombination to deliver the oxazinane ring of secu’amamine D (**8**). Altogether, these studies highlight how single-atom skeletal editing logic can both improve our understanding of biosynthetic pathways and inspire biomimetic methods for late-stage alkaloid diversification.

Oxygen-atom insertion strategies have also been applied in chemoenzymatic synthesis, for example in the synthesis of alchivemycin A (**10**) by Lei and co-workers (Scheme 1C).^42^ In this work, a flavin adenine dinucleotide-dependent enzyme AvmO1 variant was optimized through rational protein engineering to AvmO1-Y282R, which was used to convert a tetramic acid containing precursor (**9**) into the 2H-tetrahydro-4,6-dioxo-1,2-oxazine present in the natural product. Operating under mild, aqueous conditions, this oxygen insertion into the lactam C–N bond completed the chemoenzymatic synthesis of alchivemycin A.

### Nitrogen-Atom Insertion

Nitrogen insertion has been known for decades, but early examples were largely carbonyl-based and offered limited functional group tolerance. The Schmidt reaction is a well-established transformation for the conversion of carbonyl compounds or alkenes into amides, nitriles, and aza-heterocycles through nitrogen insertion via C–C or C–H bond cleavage. In the 1957 synthesis of (+)-muscopyridine (**14**) by Büchi and co-workers, an intermolecular Schmidt reaction enabled the conversion of a cyclopentene precursor (**11**) into a tetrahydropyridine containing product (**12**) (Scheme 2A).^43^ The nitrogen-atom insertion was coupled with an aromatization reaction to access the pyridine ring present in the natural product.

**Scheme 2:**
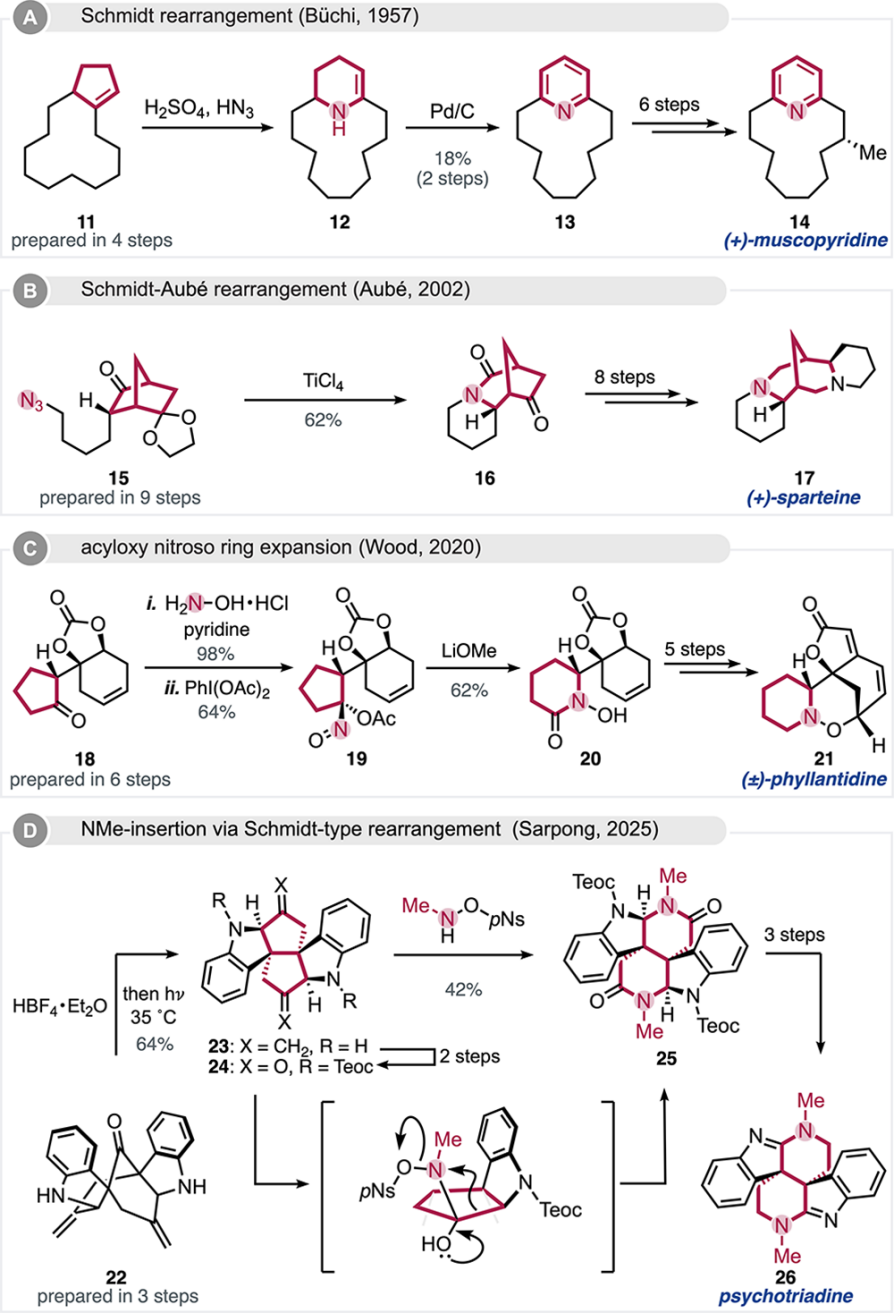
Representative Examples of Nitrogen-Atom Insertion

The Aubé modification to the Schmidt rearrangement enables intramolecular reactivity on cyclic ketone scaffolds, analogous to the Baeyer-Villiger oxidation, providing a direct route to bicyclic lactams from simple precursors. The efficacy of this transformation has unsurprisingly resulted in its application to the synthesis of many complex natural products.^43–47^ For example, Aubé’s synthesis of (+)-sparteine (**17**)^44^ showcases the power of nitrogen insertion under mild conditions, enabling the first enantioselective synthesis of this alkaloid (Scheme 2B). The Schmidt-Aubé reaction has also been employed as a part of cascade sequences with other transformations, such as a Diels-Alder cycloaddition in Aubé and co-workers’ synthesis of stenine.^47^

While azides have been regarded as reliable nitrogen sources in Schmidt-type rearrangements, other less common nitrogen donors, such as nitroso moieties and hydroxylamine derivatives, have also been used to achieve similar nitrogen insertions. In Wood’s total synthesis of (±)-phyllantidine (**21**), a nitroso-mediated ring expansion was employed to access a *N*-hydroxypiperidin-2-one motif (Scheme 2C).^46^ The five-membered nitroso precursor (**19**) was prepared from a readily available cyclopentanone (**18**) through oxime formation and oxidation. Subsequent treatment of **19** with lithium methoxide provided the ring-expanded *N*-hydroxypiperidin-2-one (**20**), bearing an *N*-hydroxy group not accessible from a conventional Schmidt-Aubé protocol. The *N*-hydroxy group was later incorporated into the oxazabicyclo[3.3.1]­nonane core. Most recently, in their synthesis of C_2_-symmetric bis­(cyclotryptamine) alkaloids, Sarpong et al. applied a Schmidt-type rearrangement to access a common intermediate that unifies their preparation of various bis­(cyclotryptamine) alkaloids (Scheme 2D).^48^ Leveraging symmetry allowed for efficient access to intermediate **24** with a congested skeleton, which set the stage for the double nitrogen insertion. To introduce the requisite skeletal nitrogen atoms, a nosyloxymethyl amine reagent triggered a Schmidt-type rearrangement that smoothly yielded the piperidinoindoline framework. This single-atom insertion strategically installed the requisite NMe groups with high regioselectivity and produced common intermediate **25** that served as the precursor to psychotriadine (**26**) and the rest of the C_2_-symmetric bis­(cyclotryptamine) alkaloid family.

### Carbon-Atom Insertion

Beyond heteroatom insertion, carbon-atom insertion into ring systems, particularly adjacent to carbonyl-based functional groups, is arguably one of the most well-established single-atom insertion strategies.^49^

Classical 1,2-migration chemistry, such as the (semi)­pinacol and benzilic acid-type rearrangement, has often been employed for ring expansion in total synthesis.^25,50–54^ Although semipinacol rearrangements lead to structural remodeling in fused ring systems, when the activated leaving group of the diol resides on an exocyclic substituent, the rearrangement can be defined as a skeletal edit. For example, in Corey’s seminal synthesis of longifolene (**29**), a semipinacol rearrangement of the mono-*p*-toluenesulfonate (**27**) was employed to achieve a ring expansion to the seven-membered ring (**28**) (Scheme 3A).^50,51^ In addition to the typical semipinacol rearrangement, thio- and aza- semipinacols or cascade processes have been employed for ring expansions in total synthesis to leverage amine and sulfide (thioethers) functionalities or ring strain release, respectively.^52,53,55,56^

**Scheme 3:**
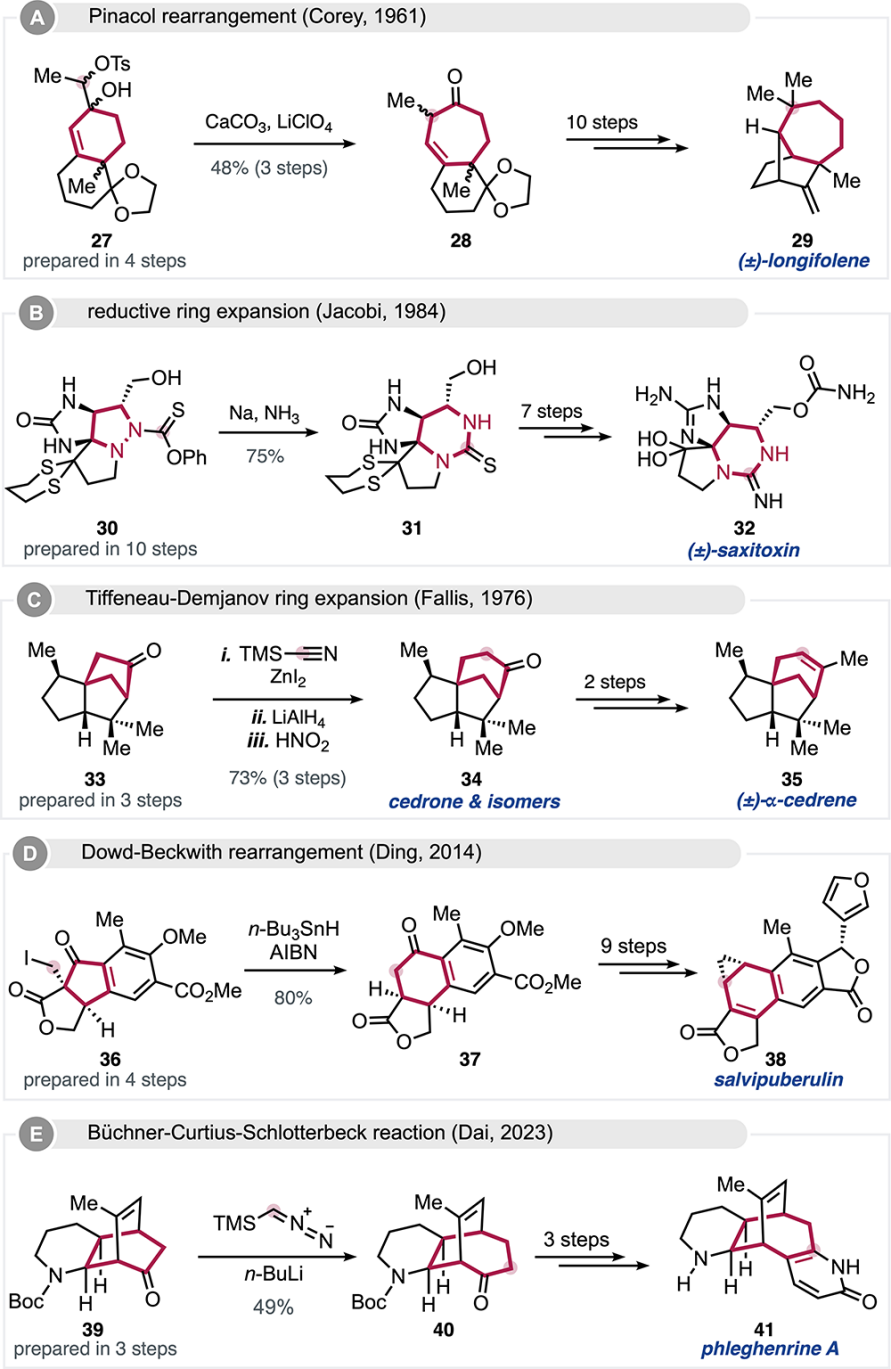
Representative Examples of Carbonyl-Based Carbon-Atom Insertion and Ring Expansion

An unusual one-carbon ring expansion was employed in Jacobi’s synthesis of saxitoxin (**32**).^57^ In this approach, a preinstalled thiocarbamate moiety enabled the reductive ring expansion of a pyrazolidine intermediate (**30**), delivering the pyrimidine present in the core of saxitoxin (Scheme 3B). The ring expansion precursor (**30**) was accessed on multigram scale through a (3 + 2) cycloaddition, which efficiently forged the pyrrolidine and pyrazolidine rings and established three contiguous stereocenters. From an aspirational standpoint, it would be powerful if this type of heterocycle expansion could be generalized and expanded to other ring systems beyond pyrazolidine.

The Tiffeneau-Demjanov rearrangement offers a reliable approach for increasing the size of amino-substituted cycloalkane and cycloalkanol systems by a single carbon, making it especially useful for accessing complex ring systems in natural product total synthesis.^58^ In the racemic synthesis of cedrene (**35**) and cedrol, Fallis and co-workers employed such a ring expansion strategy to access the bridged ring system (Scheme 3C).^59^ The early steps of the synthesis featured an intramolecular Diels-Alder reaction of an alkylated cyclopentadiene, followed by oxidation to furnish the ketone precursor (**33**) required for the ring expansion. A three-step sequence was then employed to realize the desired one-carbon insertion with relatively low efficiency. Treatment of the ketone precursor with TMSCN and ZnI_2_ generated the corresponding trimethylsilylated cyanohydrin, which was then reduced with lithium aluminum hydride to afford a primary amine. Subsequent treatment with nitrous acid afforded the 5,6-fused ring system of cedrone (**34**), which was further elaborated to (±)-cedrene (**35**).

A mechanistically distinct yet conceptually analogous approach for a one-carbon ring expansion is the Dowd-Beckwith reaction, which provides a carbocation-free, radical-based alternative. This method proceeds via the generation of an alkyl radical, which engages a carbonyl group to form an alkoxy radical, resulting in ring expansion through β-scission. Although this approach typically requires suitably activated precursors (i.e., alkyl halides), it allows for flexible and efficient synthesis of complex carbocycles and has been applied in numerous total syntheses.^60^ In 2014, Ding et al. leveraged the Dowd-Beckwith rearrangement in a diastereoselective total synthesis of salvipuberulin (**38**) and four biosynthetically related diterpenoids (Scheme 3D).^61^ More specifically, the indanone iodide precursor (**36**) was prepared through an intramolecular Heck reaction followed by iodomethylation. A subsequent Dowd-Beckwith ring expansion constructed the desired 5,6-bicyclic framework. This transformation set the stage for the installation of a cyclopropane unit, which in turn enabled the 6π electrocyclic ring opening of the benzonorcaradiene moiety to furnish the cycloheptatriene core of the biosynthetically related diterpenoid dugesin B.

The Büchner-Curtius-Schlotterbeck reaction remains a reliable strategy for achieving one-carbon insertion with various diazoalkanes especially diazomethanes, providing a direct route to homologated or expanded ring systems in a single operation (Scheme 3E). This reactivity has been employed across a range of natural product syntheses.^58,62–67^ In the synthesis of the phleghenrines by Dai and co-workers, a rigid bicyclo[2.2.2]­octenone (**39**), readily accessed via an inverse electron-demand Diels-Alder cycloaddition, underwent a Büchner-Curtius-Schlotterbeck reaction, delivering the corresponding bicyclo[3.2.2]­nonenone framework (**40**) characteristic of phleghenrine A (**41**) and C.^66^ The carbon-atom insertion allowed for a more rapid construction of the bicyclo[3.2.2]­nonenone skeleton, which would have been more synthetically challenging to access otherwise.

As the field of single-atom skeletal editing continues to evolve, there is growing interest in more generalizable one-carbon insertion strategies, especially into aromatic systems. Carbenes have become increasingly useful single-atom carbon sources for skeletal editing. Among carbon insertion methods into aromatic systems, the Büchner ring expansion is one of the most well-established and widely utilized reactions. An intramolecular adaptation of this chemistry appears in the synthesis of daphenylline (**44**) by Sarpong and co-workers, where a Büchner-type carbene insertion into the acenaphthyl moiety (**42**) followed by a 6π electrocyclic ring opening and reduction delivered cycloheptadiene (**43**) (Scheme 4A).^68^ This two-step transformation demonstrates how the combination of carbene insertion and pericyclic ring-opening can rapidly build complexity from readily available aromatic starting materials.

**Scheme 4:**
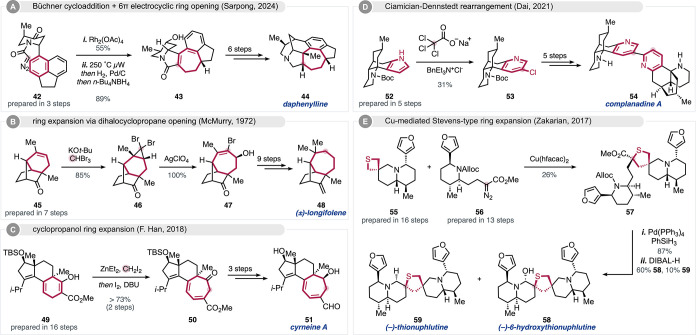
Representative Examples of Carbene-Based Carbon-Atom Insertion and Ring Expansion

(Dihalo)­carbene- and cyclopropane-based intermolecular ring expansion reactions have also found widespread utility in total synthesis, enabling strategic carbene-derived carbon-insertions into highly congested frameworks.^69–73^ A hallmark example of this reactivity is found in the synthesis of longifolene (**48**) by McMurry et al., where *gem*-dibromocyclopropanation followed by silver-ion-promoted ring expansion furnished a key allylic alcohol (**47**) and established the bridged 6–7 ring system of longifolene (Scheme 4B).^74^ Crucially, the resulting allylic alcohol set the stage for the installation of an additional methyl group found in longifolene. In addition to carbene cycloaddition, other cyclopropane formations coupled with ring fragmentations have also been reported to achieve a ring expansion in synthesis.^75–77^

Cyclopropanation followed by scission of the strained three-membered ring has been further expanded to edit in macrocyclic ring systems. Zercher’s use of β-keto esters, which have high enolic content, demonstrates that the cyclopropanation followed by fragmentation approach can be achieved in a single step and effectively modify macrocyclic systems.^78^ An example of the Zercher-type cyclopropane-based strategy for single-atom carbon insertion using β-ketoesters was demonstrated by F. Han and co-workers in their syntheses of cyrneine A (**51**) and B (Scheme 4C).^79^ In this approach, the *in situ*-generated carbene serves as a one-carbon unit, and selective cyclopropanol C–C bond cleavage enabled a formal carbon insertion event en route to the desired 5/6/7 tricyclic system.

Carbenes have also found utility for the skeletal editing of heterocycles such as pyrroles and indoles. The Ciamician-Dennstedt rearrangement is one of the few modes of reactivity to date that accomplishes a one-step carbon insertion through a *gem*-dihalocyclopropane intermediate into heteroaromatics. However, the application of the Ciamician-Dennstedt reaction in synthesis has been limited. In the synthesis of complanadine A (**54**) by Dai and co-workers (Scheme 4D),^80^ the synthetic design relied on a Ciamician-Dennstedt to convert a pyrrole (**52**) into the corresponding pyridine (**53**) while simultaneously installing a chlorine handle on the pyridine nucleus to enable late-stage diversification via cross-coupling reactions.^81^ As opposed to the electron deficient pyridine, the nucleophilicity of the electron-rich pyrrole precursor was essential for an earlier stage key C–C bond formation, necessitating the late-stage pyrrole-to-pyridine conversion. Mechanistically, of the two halides present in dihalocarbenes for the Ciamician-Dennstedt reaction, only one is required as a nucleofuge for the fragmentation step, suggesting that the second halide could be replaced with a different functional group to expand the scope of carbene-based carbon insertions, as demonstrated in Levin’s modification of the Ciamician-Dennstedt rearrangement.^82^

As a complement to cyclopropanation-based transformations, carbenes have also been utilized to expand saturated heterocycles, for example, through direct C–S insertion. Zakarian and co-workers demonstrated that carbene insertion into thietanes can effectively construct the central spirothiolane ring system of thioalkaloids.^83^ Late in the synthetic sequence, the thietane precursor (**55**), prepared from a common piperidine intermediate, underwent a copper-mediated Stevens-type ring expansion to furnish the corresponding amino ester (**57**), which was subsequently reduced to provide (−)-6-hydroxythionuphlutine (**58**) and (−)-thionuphlutine (**59**) (Scheme 4E). These transformations underscore the expanding scope of carbene-mediated insertions and point toward the possibility of broader application across other heterocyclic systems.

### Aspirational Goals for Single-Atom Insertion Strategies

To date, noncarbonyl-based methods for single-atom insertion and ring expansion remain relatively limited in scope. Insertions of heteroatoms often require an adjacent carbonyl functionality, whereas carbene reactivity primarily engages π-systems to enable facile insertion of carbon atoms into heteroaromatics and all-carbon cyclic frameworks. Nonetheless, recent advances suggest emerging momentum in this space. For example, Levin and co-workers reported a ring expansion strategy in which pyrazoles and indazoles are converted to pyrimidines and quinazolines, respectively, via carbon insertion into N–N bonds.^84^ In a similar light, methods to efficiently accomplish direct single-atom insertions into (hetero)­aromatic moieties have garnered attention in the synthetic community, particularly due to the high prevalence of nitrogen-containing heterocycles in medicinal compounds and natural products.^85^ While one-carbon insertions into indoles have precedent, analogous nitrogen-atom insertions had not been realized until a report by Morandi and co-workers. By leveraging an *in situ*-generated iodo-nitrene reagent,^86,87^ direct nitrogen insertion into indoles was achieved to construct quinazolines, a valuable class of N,N-heterocycles. Recently, Maseras and Suero et al. reported the only atroposelective single-carbon insertion to date, building atropochiral quinolines from 3-aryl indoles through a Rh-catalyzed enantioselective ring expansion.^88^ While these methods hold great potential, they have yet to be applied to natural product synthesis.

Despite many notable advances, the broader aspiration for general, functional group tolerant methods for single-atom insertion at nonactivated positions which can enable total synthesis remains largely unrealized. One can envision temporarily functionalizing heteroatoms (e.g., nitrogen or sulfur) to enable directed single-atom insertion. Ideally, editing of hydrocarbon frameworks devoid of preinstalled carbonyl groups or other traditionally activating functionalities would be possible. In this context, single-atom insertions into unstrained and unfunctionalized C­(sp^3^)–C­(sp^3^) bonds are arguably the holy grail, representing a frontier that, if realized, could significantly expand the synthetic possibilities of skeletal editing. Equally compelling are stereochemically defined or more functionalized atom insertions and editing strategies that can repurpose chiral pool molecules to streamline access to otherwise inaccessible natural product frameworks. The emphasis on nitrogen, oxygen, and carbon atoms reflects their prevalence in natural products, but looking ahead, the broader aspiration is for skeletal editing methods to expand to other heteroatoms, such as sulfur, boron, or phosphorus, enabling new synthetic opportunities both within and beyond total synthesis.

## Deletions and Ring Contractions

Single-atom deletions and ring contractions often enable facile construction of highly strained or sterically congested natural product cores. A strategically placed atom, such as carbon, sulfur, oxygen, or nitrogen, can be used to facilitate ring formation, functionalization, or fragment coupling. The subsequent deletion of these atoms is a powerful late-stage transformation that provides access to a range of cyclic motifs.

### Carbon-Atom Deletion

Classical carbonyl reactivity has long been leveraged to contract ring systems by one carbon atom (Scheme 5). As a complement to its application in ring expansions, the Tiffeneau-Demjanov rearrangement was employed by Woodward et al. on an α-amino diol precursor to generate an exocyclic aldehyde-bearing cyclopentanol product for the synthesis of (±)-prostaglandin F2α (**3**) (Scheme 5A).^89^ This synthesis began with *cis*-cyclohexane-1,3,5-triol which enabled stereochemical control in the preparation of the α-amino diol (**60**). The subsequent ring contraction then gave access to the four contiguous stereocenters in the cyclopentane core of (±)-prostaglandin F2α, directly addressing the key stereochemical challenge inherent to its synthesis. While the carbon atom was ultimately not deleted from the molecular scaffold, the excised aldehyde (**61**) provided a functional handle for Wittig olefination to install the requisite substituent en route to the natural product. The Wolff rearrangement has also been employed for the ring contraction of α-diazoketone precursors to generate exocyclic carbonyl groups, such as in the Oppolzer synthesis of (±)-isocomene (**64**) (Scheme 5B).^90–92^ While subsequent deletion of the carbon atoms excised via Wolff rearrangement is less common, the resulting exocyclic carbonyl groups have served as functional handles in a variety of natural product syntheses.

**Scheme 5:**
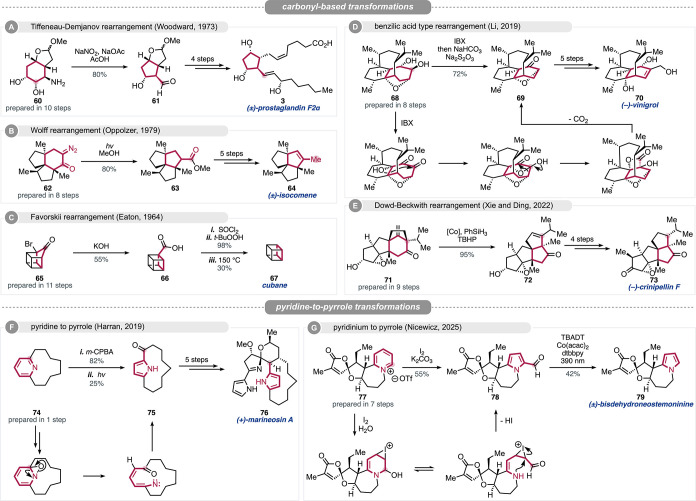
Representative Examples of Carbon-Atom Deletion and Ring Contraction

On the other hand, several types of carbonyl ring contraction reactions have been coupled with decarboxylation sequences to accomplish the overall deletion of a carbon atom from the molecular skeleton. In 1964, Eaton and co-workers leveraged two Favorskii rearrangements of α-bromoketone precursors (including **65**) to afford four out of the six cyclobutane rings of cubane (**67**) (Scheme 5C).^93,94^ The resulting exocyclic carboxylic acids (such as **66**) were then sequentially removed by conversion to the corresponding *t*-butyl peresters followed by thermal radical fragmentation, accomplishing a formal carbon deletion from the cyclopentanone precursors, albeit through multistep sequences. Revisiting these classical carbonyl rearrangements in combination with decarboxylation methods developed recently should enable more concise methods for carbon-atom deletions.

Benzilic acid type rearrangements of cyclic diketone precursors have also historically been used to achieve ring contractions through a 1,2-alkyl shift.^95–97^ In 2019, Li et al. coupled this type of reactivity with an *in situ* decarboxylation to achieve a one-step carbon deletion in their synthesis of (−)-vinigrol (**70**) (Scheme 5D).^98^ More specifically, the cycloheptane diol precursor (**68**)—initially prepared through an oxidopyrylium ylide (5 + 2) cycloaddition—underwent oxidation to generate a hydroxy 1,2-diketone upon treatment with IBX. Basic workup led to an intramolecular attack by the newly installed hydroxy group on the less hindered carbonyl to yield an oxetanone intermediate. Analogous to benzilic acid-type rearrangements, this oxetanone was proposed to undergo a 1,2-alkyl shift generating an unstable β-lactone fused cyclohexanol. Spontaneous decarboxylation of the β-lactone followed by keto-enol tautomerization generated the desired cyclohexanone product (**69**), which was elaborated to (−)-vinigrol in five steps.

The Dowd-Beckwith rearrangement also enables ring contraction of cyclic carbonyl compounds. However, proceeding through a radical-based mechanism, this transformation results in the extrusion of a carbon α-to-carbonyl instead of the carbonyl carbon. This mechanistic difference means that the ring contracted product is not amenable to subsequent decarboxylation to result in a net carbon deletion, but this does not restrict its application to synthesis. In 2022, Xie and Ding et al. leveraged a Dowd-Beckwith rearrangement in a divergent total synthesis of eight tetraquinane natural products, including (−)-crinipellins A–F and (−)-dihydrocrinipellins A and B (Scheme 5E).^99^ The isopropyl group found in these natural products was originally introduced as a substituent on a functionalized benzaldehyde precursor, which enabled the construction of a 5/5/6/5 tetracyclic precursor (**71**) via a diastereocontrolled oxidative dearomatization-induced (ODI) (5 + 2) cascade. Unlike typical applications of the Dowd-Beckwith rearrangement, which require preinstallation of an alkyl halide radical handle, the 5/5/6/5 tetracyclic precursor then underwent metal-catalyzed hydrogen atom transfer (MHAT) to generate the requisite β-radical. This transformation forged the highly strained tetraquinane core (**72**) of the natural products in a single step, demonstrating the growing power and flexibility of radical-based skeletal editing in complex molecule synthesis. Newly emerging strategies for carbon deletion with high potential for future applications are focused on the transformations of N-containing heterocycles, such as pyridines. Pyridines are versatile starting points for functionalization and have been shown to undergo ring contraction through various mechanisms to form pyrroles. In the synthesis of (+)-marineosin A (**76**), Harran and co-workers leveraged the photorearrangement of a pyridinophane N-oxide, prepared in one step from a pyridinophane precursor (**74**) to access the ring contracted ketopyrrolophane product (**75**) (Scheme 5F).^100^ The rearrangement was proposed to proceed via an oxaziridine intermediate followed by subsequent nitrene formation and formal C–H insertion. However, the yield was significantly limited by the formation of side products through secondary photo processes of the intermediates as well as the desired product. This ring contraction strategy enabled Harran et al. to start the synthesis from 2,6-dichloropyridine, employing a Kumada cross coupling to rapidly access the symmetric pyridinophane. Related to this work and similar transformations,^101,102^ Levin et al. later reported an efficient variation of this method for the multistep deletion of carbon atoms from pyridines and other azaarenes through controlled irradiation with 390 nm light, which minimized side reactions.^103^

A complementary approach for pyridine-to-pyrrole conversion is through an iodine-mediated rearrangement of pyridinium salts to give 2-formylpyrroles.^104^ The application of this methodology on late stage intermediates was recently demonstrated by Nicewicz and co-workers in their 2025 synthesis of several stemoamide alkaloids (Scheme 5G).^105^ Using a pyridine in the early stage of the synthesis enabled the researchers to rapidly construct the spirobutenolide ring system through a series of photoredox transformations, overcoming the reactivity limitations of using an electron-rich pyrrole. Following the formation of the 5/7/6-pyridinium salt precursor (**77**) via a Mitsunobu reaction, iodine-mediated ring contraction afforded the 2-formylpyrrole product (**78**). In this example, the skeletal edit was particularly enabling as divergent modifications of the resulting formyl group provided access to three stemoamide alkaloid natural products. It is additionally worth noting that a subsequent metallophotoredox decarbonylation completed the synthesis of (±)-bisdehydroneostemoninine (**79**) via a two-step overall pyridine-to-pyrrole one-carbon deletion skeletal edit.

### Sulfur-Atom Deletion

In addition to carbon-atom deletion, there are various established protocols for heteroatom deletion which have been applied to total syntheses. Sulfur and other heteroatoms are typically more nucleophilic than aliphatic carbons and thus serve as excellent linker atoms for ring closure. As such, strategies that extrude heteroatoms attendant with C–C bond formation are extremely powerful for the synthesis of difficult-to-access carbocyclic systems. Perhaps the most well-known example of this kind of reactivity is the Eschenmoser sulfide contraction, which was first developed for the synthesis of Vitamin B_12_ (**82**) carried out jointly by the laboratories of Eschenmoser and Woodward in 1973 (Scheme 6A).^106,107^ Utilized three times throughout the synthesis, this sulfur deletion strategy enabled the construction of several critical C–C bonds and, ultimately, the corrin core (**81**) of the natural product. Another well-known method for sulfur extrusion is the Ramberg-Bäcklund reaction, which transforms α-halosulfone or α-halosulfoxide precursors into alkene products. In their 2016 synthesis of a natural ladderane phospholipid (**85**), Gonzalez-Martinez, Boxer, Burns and co-workers constructed the key bicyclo[2.2.0]­hexene (**84**) building block present in both ladderane tails through a Ramberg-Bäcklund reaction (Scheme 6B).^108^ Because this methodology requires preactivation of the sulfur atom by installing an α-halogen or through oxidation to the sulfone or sulfoxide, it is often employed as a multistep protocol for sulfur deletion.^108,109^

**Scheme 6:**
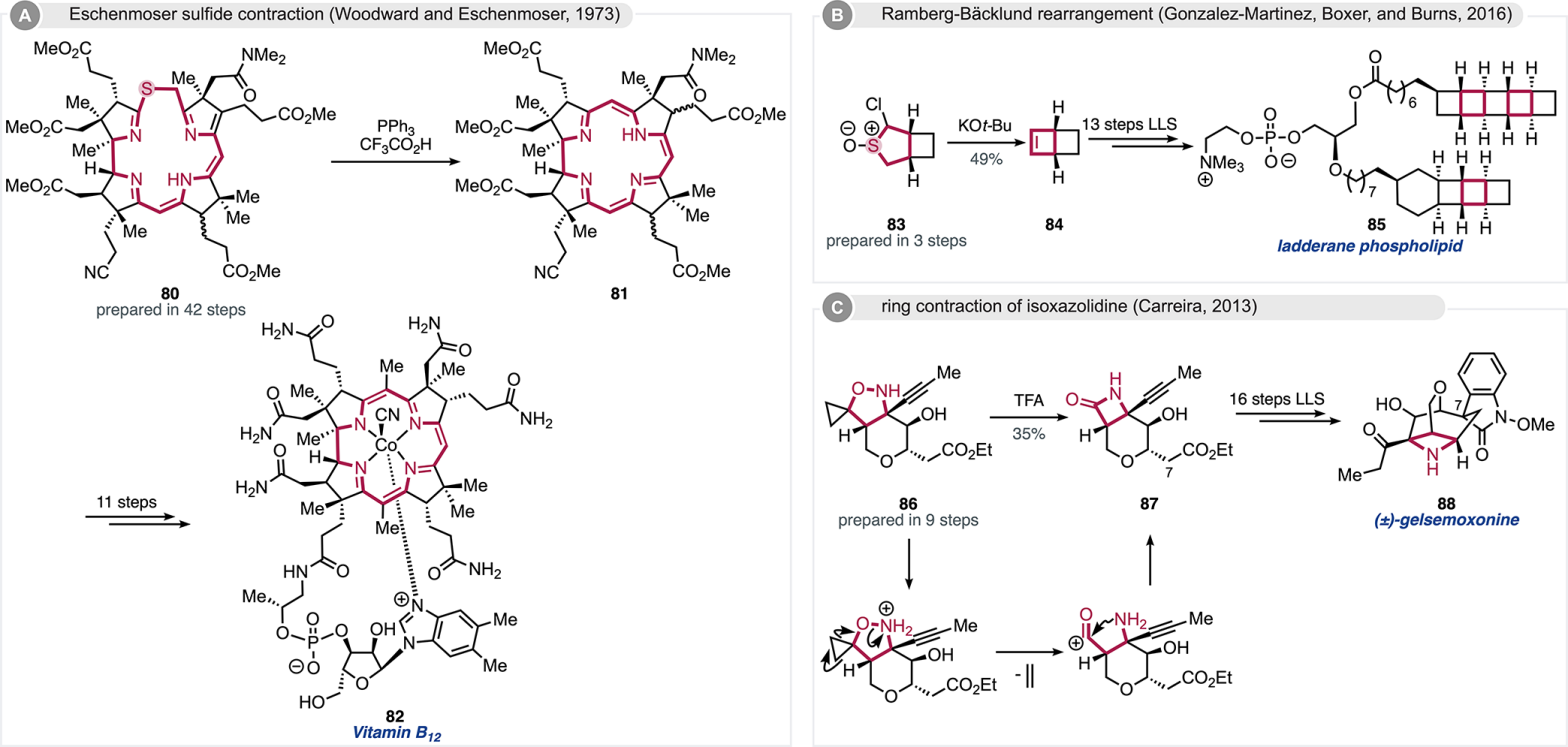
Representative Examples of Chalcogen-Atom Deletion

### Oxygen-Atom Deletion

In contrast to oxygen-atom insertion, methods for the removal of oxygen atoms from ring systems are underdeveloped. In 2000, Cordero et al. reported that spirocyclopropane isoxazolidines could furnish β-lactams through acid-mediated oxygen-atom ring contraction.^110^ Mechanistically, it is proposed that this transformation proceeds through the concerted rupture of the N–O bond and cyclopropane to release ethylene.^111^ This method has since been employed in Carreira and co-workers’ synthesis of gelsemoxonine (**88**) (Scheme 6C).^112^ The synthetic design relied on late-stage construction of the oxindole moiety by arylation at C7. While conventional approaches for β-lactam synthesis did not enable access to the desired key intermediate (**87**), the ring contraction of spirocyclopropane isoxazolidine (**86**) efficiently formed the azetidine ring present in the natural product core. The extruded oxygen atom was later removed following a downstream Petasis olefination. Although this ring contraction method is useful for β-lactam or multistep azetidine synthesis, there is still a need for more generalizable oxygen-atom deletions and ring contractions.

### Nitrogen-Atom Deletion

Modern methods have focused on the deletion of nitrogen atoms from saturated and unsaturated heterocyclic systems. In their 1993 synthesis of *d,l*- and *meso*-isochrysohermidin (**91**), Boger and co-workers reported the reductive net nitrogen deletion from a dipyridazine precursor (**89**), expediently accessed using an inverse-demand Diels-Alder cycloaddition, to form the dipyrrole core (**90**) of the natural product (Scheme 7A).^113^ Mechanistically, this transformation proceeded by zinc reduction of the pyridazine moiety to the corresponding 1,4-dihydro-1,2-diazine followed by reductive cleavage of the N–N bond to give an acyclic imine. Ring contracting cyclization into the imine and rearomatization through the loss of ammonia completed a formal deletion of a nitrogen atom from the dipyridazine scaffold. While the Kornfeld-Boger reductive ring contraction reaction has enabled several other syntheses, its application is limited to pyridazine scaffolds with substituents that are tolerant of the strongly reducing conditions required for the transformation.^114–120^ Nonetheless, this work has served as the inspiration for novel synthetic methods to accomplish diazine-to-azole transformations.^121,122^ Analogous mechanistic pathways, proceeding through a ring contracting cyclization of an acyclic imine and rearomatizing elimination can be found in various modern atom deletion and transmutation methodologies that rely on addition of a nucleophile, ring opening, and ring closure (ANRORC) mechanisms.^123^

**Scheme 7:**
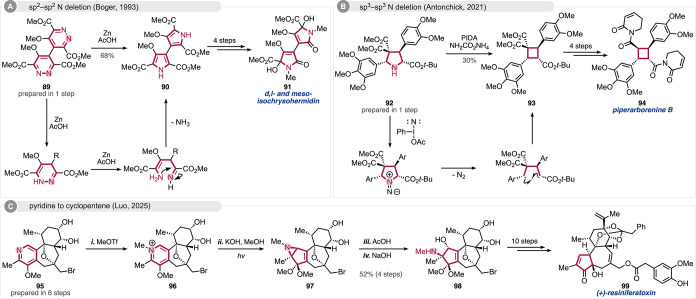
Representative Examples of Nitrogen-Atom Deletion

For saturated nitrogen-containing ring systems, Antonchick et al. reported a method for the deletion of a nitrogen atom from pyrrolidine precursors (**92**), readily synthesized in one step using Ag-catalyzed (3 + 2) cycloaddition, to form cyclobutane products. This methodology was applied to the formal synthesis of piperarborenine B (**94**) (Scheme 7B).^124^ The transformation is enabled by *in situ* generation of an iodonitrene species that affords the key reactive 1,1-diazene intermediate through electrophilic amination. Nitrogen extrusion furnishes a 1,4-biradical species that undergoes intramolecular C­(sp^3^)–C­(sp^3^) bond formation to yield the desired cyclobutane product (**93**). Although this is a rare example for this type of nitrogen deletion in total synthesis, the principle of activating a secondary amine via electrophilic amination and subsequent nitrogen extrusion has inspired the development of synthetic methodologies that could provide inspiration for future total syntheses, most notably by the laboratories of Levin and H. Lu.^125–131^

From an aspirational standpoint, the use of nitrogen containing heterocycles—which have been readily explored in a skeletal editing context—to access saturated cycloalkane moieties has the potential to be a very powerful tool for total synthesis. To this end, Luo and co-workers recently leveraged a multistep ring contraction of a pyridine (**95**) to access an amino cyclopentene product (**98**) that underwent downstream deamination to furnish the cyclopentene moiety of (+)-resiniferatoxin (**99**) (Scheme 7C).^132^ Prior to ring contraction, the rich chemistry of pyridines enabled the efficient construction of the central seven-membered ring of the natural product. Mechanistically, the ring contraction is believed to proceed through the formation of a pyridinium salt (**96**) and subsequent photorearrangement^133^ to access a modestly stable aziridine fused cyclopentene (**97**), which was derivatized in two steps prior to purification. After an additional five steps, the exocyclic amine was lost to ultimately complete the nitrogen “deletion” from the original pyridine scaffold. While this “deletion” occurs over multiple steps with modifications to the molecule occurring in between, it nonetheless serves as an inspiration for the future development of methodologies to directly access cyclopentene and other cycloalkane rings from heterocycles via dearomative heteroatom deletion.

### Linchpin Strategies

Although not contained within the core ring system and thus not classified as skeletal edits in this Perspective, the deletion of acyclic linker atoms or linchpins are also enabling strategies for the construction of sterically congested or otherwise hard to access C–C bonds. Carbonyl groups, specifically (C=O), serve as model linchpins due to the ease of stereoselective functionalization at the α-position through well-established enolate chemistry. As such, methods for stereoretentive decarbonylation can enable the efficient formation of contiguous quaternary carbon centers present in challenging natural product scaffolds. In 2021, Houk, Garcia-Garibay, Garg and co-workers used this strategy to form the central C–C bond of psychotriadine (**26**) (Scheme 8A).^134^ Starting from a hexasubstituted ketone precursor (**100**), solid state photodecarbonylation and subsequent lactam deprotection gave access to the two contiguous all carbon stereocenters present in the natural product. Aside from monocyclic examples by Garcia-Garibay and co-workers, photodecarbonylations have seen limited applications in cyclic scaffolds, especially for late-stage carbon deletion in the total synthesis of more complex ring systems.^135,136^ In the recent synthesis of the C_2_-symmetric bis­(cyclotryptamine) alkaloids, Sarpong et al. employed a related solution-state decarbonylative deletion of a bridging carbon atom from a [3.3.1]-bicyclic intermediate (**22**, Scheme 2D) to generate a *cis*-fused 5,5-bicyclic precursor (**23**) that was primed for *N*-insertion.^48^ While this example is of a linchpin deletion for skeletal contraction, it demonstrates the potential for photodecarbonylation in the solution-state on more complex scaffolds.

**Scheme 8:**
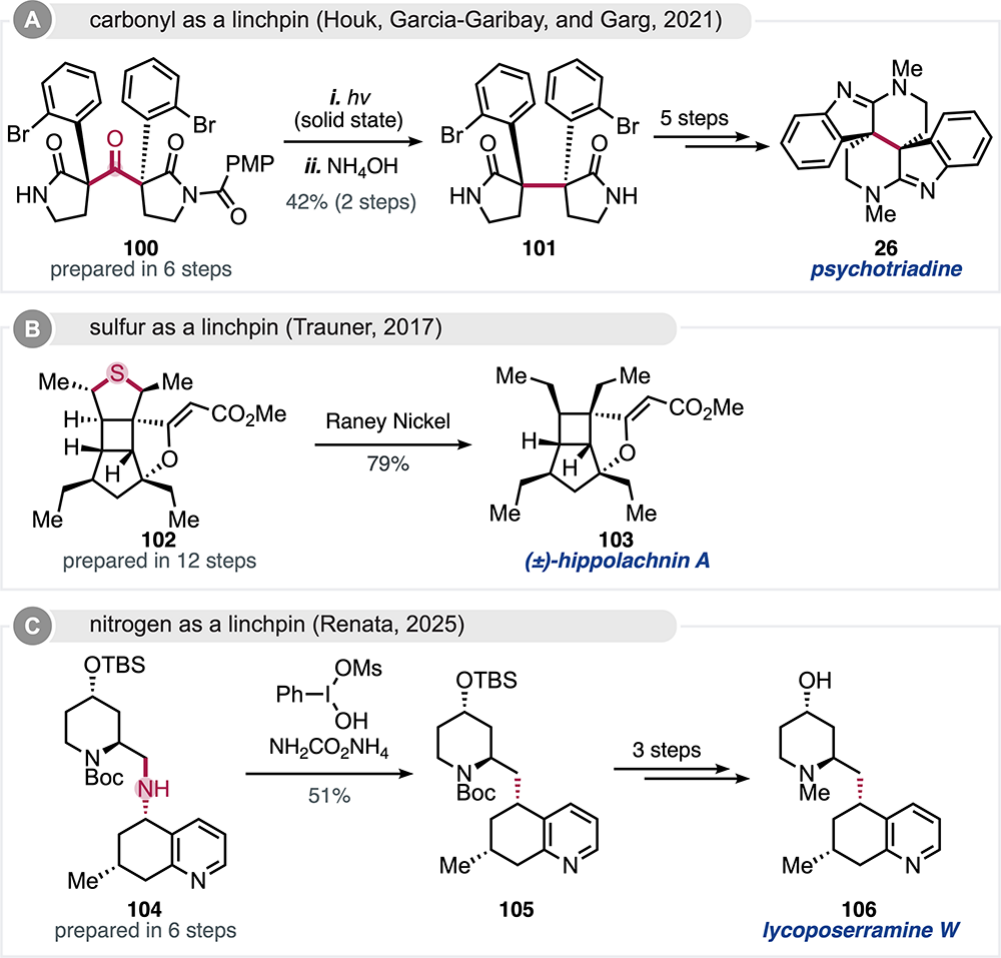
Representative Examples of Linchpin Deletion

The deletion of heteroatoms from ring systems has also been extended to sulfur and nitrogen linchpins. The Eschenmoser sulfide contraction and Ramberg-Bäcklund reaction, in addition to the cyclic examples previously discussed, have been applied to the deletion of sulfur linchpins to furnish acyclic C–C bonds in various total syntheses.^106,107,137^ Complementary to these methods is the Mozingo reduction in which C–S bonds are reduced to C–H bonds through a formal deletion of the sulfur atom.^68,138–142^ In the synthesis of (±)-hippolachnin A (**103**) by Trauner and co-workers, a combination of an alkene-thiocarbonyl ylide 1,3-dipolar cycloaddition and a late-stage Mozingo reduction enabled the introduction of the two exocyclic ethyl substituents (Scheme 8B).^138^ The application of this sulfur linchpin deletion strategy overcame diastereoselectivity issues inherent in ethyl installation using classical carbonyl alkylation chemistry.

A nitrogen linchpin deletion strategy, related to Antonchick’s hypervalent iodine-based method, was also used by Renata et al. in their 2025 synthesis of lycoposerramine W (**106**) (Scheme 8C).^143^ The tetrahydroquinoline moiety and the piperidine fragment of the target natural products were coupled through a reductive amination followed by removal of the nitrogen linker. This application of a late-stage nitrogen linchpin deletion enabled an efficient construction of the natural product in a stereocontrolled manner and expanded this methodology for nitrogen extrusion to furnish an acyclic C­(sp^3^)–C­(sp^3^) bond.

### Aspirational Goals for Single-Atom Deletion Strategies

In an ideal skeletal edit, chemists would be able to fully delete an atom from a molecular scaffold in a single late-stage step, but broadly applicable methods for doing so are still quite limited. Methods that have been employed for carbon deletion often rely on multiple steps featuring an initial ring contraction and subsequent functionalization of the extracted carbonyl functionality or deletion via decarboxylation. Sulfur extrusion methods are limited and furnish 1,3-dicarbonyl, alkene, or fully reduced alkane (C–S to C–H) products. Deletion methodologies for nitrogen-containing saturated and unsaturated heterocycles are perhaps the most well explored in modern skeletal editing, however many of these newer methods are restricted to substrates with stabilizing groups and have yet to be applied to natural product synthesis.

As the field of skeletal editing evolves to meet the needs of complex molecule synthesis, there are several transformations to aspire to. For example, the expansion of modern deletion techniques to unfunctionalized carbons, particularly to enable the removal of methylene groups from cyclic scaffolds, would significantly impact the way syntheses are designed. Furthermore, complementary methods for heteroatom deletion—in the absence of stabilizing groups—to generate unfunctionalized C­(sp^3^)–C­(sp^3^) bonds can broaden the application of heteroatom linchpins in acyclic and cyclic systems. The development of strategies for facile deletion of atoms from highly complex starting materials—such as chiral pool terpenes, carbohydrates, or peptides—would provide efficient access to new chemical space. More specifically, methods for oxygen deletion are underexplored and would enable chemists to access cyclopentane and cyclobutane containing scaffolds from highly oxidized carbohydrate starting materials.

## Transmutations

Single-atom transmutation describes the exchange of atoms within a core skeleton—most valuable at a late stage in a synthetic sequence. This skeletal edit enables facile functionalization or construction of core ring systems in the presence of a chemically inert or potentially advantageous core atom, which can then be converted to the desired and often more reactive or challenging to construct, cyclic scaffold resident in the natural product.

Methodologies to efficiently accomplish such atom swapping have recently gained popularity within the synthetic community, but their application in total synthesis has been very sporadic. However, even in the classical era of total synthesis, chemists were strategically swapping core atoms to access complex natural product scaffolds. The Diels-Alder reaction of 2-pyrones (**107**) and subsequent retro-[4 + 2] to release carbon dioxide is a formal multiatom swap in which the carbons of the dienophile replace the carbon and oxygen of the lactone (Scheme 9A). This strategy has been employed with a range of dienophiles in various total syntheses, including that of rufescine (**109**) and related azafluoranthene alkaloids by Boger and co-workers.^144^ When performed in an intramolecular fashion, this formal oxygen-to-carbon transmutation has been used to generate macrocycles containing bent aromatic rings, such as in Baran’s synthesis of (−)-haouamine A.^145^ Although this mode of reactivity technically involves a multiatom swap, it demonstrates the potential of such an editing strategy in natural product synthesis.

**Scheme 9:**
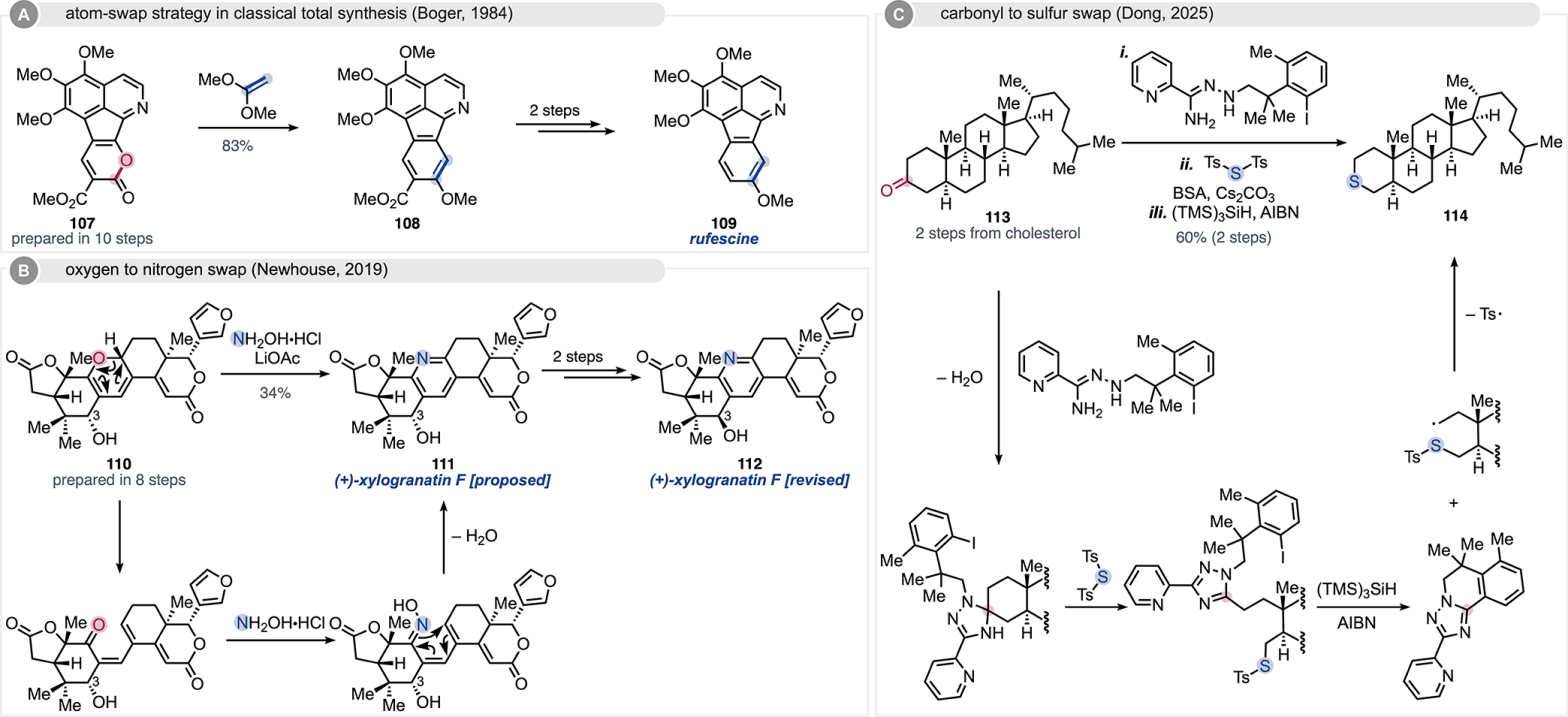
Representative Examples of Single-Atom Transmutations

A more direct single-atom transmutation was demonstrated in the Newhouse 2019 total synthesis of xylogranatin F (**112**) and related bislactone limonoid alkaloid natural products (Scheme 9B).^146^ In this work, the investigators achieved an oxygen-to-nitrogen transmutation by a late-stage pyran-to-pyridine (**110** → **111**) transformation via a tandem sequence of retro-oxa-6π electrocyclization, condensation of hydroxylamine, aza-6π electrocyclization, and elimination of a water molecule. The pyran precursor was initially formed through a selective 1,3-diketone differentiation, which enabled access to the requisite benzylic oxidation state at C3. Efforts to accomplish late-stage benzylic oxidation had been unsuccessful with the pyridine already in place, but this oxygen-to-nitrogen transmutation strategy provided an elegant solution.

### Aspirational Goals for Single-Atom Transmutation Strategies

Despite the limited number of applications in total synthesis to date, there are a number of recent methodologies emerging for single-atom transmutations. For example, Dong and co-workers reported a multistep carbonyl-to-sulfur swap that has been demonstrated on a derivative of cholesterol (**113**) (Scheme 9C).^147^ Koh et al. have reported the conversion of oxetanes to azetidines or thietanes.^148^ Various groups have also disclosed transmutation-based methods for the interconversion of various aromatic heterocycles, including pyridine-to-pyridazine (C → N),^149^ furan-to-pyrrole (O → N),^150^ isoquinoline-to-naphthalene (N → C),^151^ and indole-to-indazole (C → N)^152^ transformations. Several methods for isotopic exchange of carbon and nitrogen atoms have also been reported.^153–156^ If applied strategically, these methods have the potential to significantly facilitate natural product total synthesis, analog generation, and biological studies.

There is a need for the development of more methods for heteroatom transmutations beyond those reported in the literature and applications to carbocycles. For example, oxygen to sulfur, nitrogen, or carbon transmutations in carbohydrate substrates would allow for rapid access to thio- and aza-sugars as well as highly oxidized carbocycles. Additionally, novel methods for single-atom transmutations on highly complex peptide and chiral pool starting materials could be synthetically enabling. For instance, a direct O → N transmutation would make the amidine analog synthesis for biologically active peptides such as vancomycin^157^ much more facile and a carbon to heteroatom swap in chiral terpene scaffolds would give rise to heterocyclic chiral pool starting materials for synthesis.

## Stereochemical Editing

Despite its synthetic utility and importance, the ability to reconfigure stereochemistry directly within an existing framework remains heavily underdeveloped. Total synthesis often demands late stage stereochemical correction, typically because early stereocontrol in complex systems is challenging, inefficient, or incompatible with downstream steps. This mismatch between high synthetic demand and a low number of existing methods for this purpose underscores the need for expanding this dimension of the skeletal editing toolbox.

In contrast to classical stereocontrol, where configuration is treated as fixed from the moment a bond is formed, stereochemical editing treats stereocenters as editable nodes. Historically, such opportunities were largely limited to activated positions (typically α-to-carbonyl groups), which often rely on thermodynamic control. However, when the desired product is not thermodynamically favored, chemists can often go to extreme measures to achieve the desired epimerization. The total synthesis of (±)-reserpine (**117**) by Woodward is illustrative (Scheme 10A).^158–160^ An unforeseen consequence of their synthetic strategy led to the undesired configuration at C3 for **115**, which needed to be corrected late in the sequence. To favor the desired epimerization, a lactone ring (not present in the final natural product) was transiently constructed. The resulting strain imparted on the hexacyclic core of the precursor reversed thermodynamic preference enabling epimerization at the ring junction followed by deconstruction of the lactone. This creative but multistep synthetic problem-solving illustrates how challenging it can be to access a less thermodynamically favored isomer, revealing both the strategic value and the historical limitations of when such stereochemical edits could be made in total synthesis. Classical examples underscore the unmet need for generalizable strategies capable of editing stereocenters in less activated environments.

**Scheme 10:**
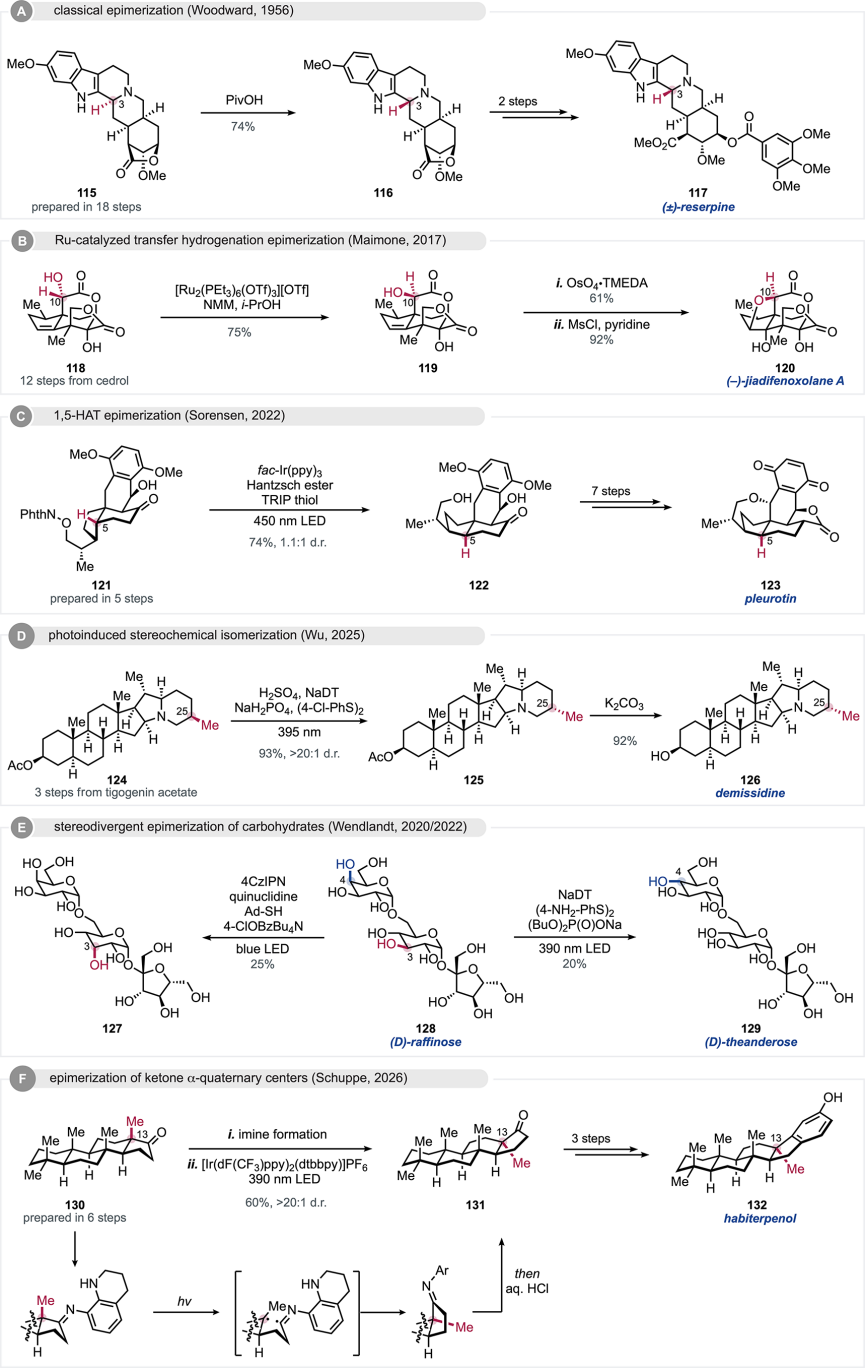
Representative Examples of Stereochemical Editing

In Maimone’s syntheses of jiadifenoxolane (**120**) and majucin,^161^ the synthetic strategy hinged on effecting a late-stage stereochemical adjustment at C10 of the hydroxy lactone intermediate (**118**) (Scheme 10B). In the synthetic sequence, late-stage α-hydroxylation from the convex face gave the undesired configuration at C10. To correct this stereochemical issue, they used a mild Ru-catalyzed transfer hydrogenation developed by Hartwig,^162^ which cleanly epimerized the stereocenter. Other methods were less desirable due to the high fragility of the substrate.

More recent approaches have turned to photocatalyzed stereochemical editing. An elegant example is in the total synthesis of pleurotin (**123**) by Sorensen and co-workers,^163^ wherein a photochemical HAT-mediated C–H epimerization was employed to change the configuration at the unactivated C5 methine stereocenter of **121** (Scheme 10C) and convert the undesired but easy-to-access *cis*-hydrindane to the desired but more strained *trans*-hydrindane encoded in pleurotin. Mechanistically, a reactive oxygen-centered radical, generated from the preactivated alcohol, initiated a downhill 1,5-hydrogen atom transfer to form a tertiary alkyl radical. Then, a sterically hindered arylthiol transfers a hydrogen atom to the alkyl radical in a kinetically controlled step to partially epimerize the C5 stereocenter. This photoredox system played an important role in establishing the correct relative stereochemistry of pleurotin’s carbon framework. More broadly, this approach highlights how stereochemical editing can address otherwise difficult stereochemical problems without redesigning the entire synthetic route.

An example of photocatalytic stereochemical editing of tertiary C–H bonds of steroids appears in the synthesis of demissidine (**126**) and other natural products by Wu et al. (Scheme 10D).^164^ Selective hydrogen atom abstraction (HAA) at C25 of tigogenin acetate (**124**) posed a significant challenge due to the lower bond dissociation energy of the more hydridic α-amino C–H bond, which is more prone to abstraction under the conditions that were used. To overcome this selectivity challenge, protonation of the amine with sulfuric acid changed the selectivity for HAA, leading to epimerization at C25 to access the desired stereoconfiguration. Following hydrolysis, the natural product demissidine (**126**) was obtained, completing a four-step synthesis. This work shows that challenging tertiary stereocenters can be selectively edited, even in the presence of weaker α-amino C–H bonds.

Photochemical methods have also been explored in the context of glycoside editing, as described in the isomerization of trisaccharides by Wendlandt et al. (Scheme 10E).^165^ Under quinuclidine-catalyzed conditions, raffinose (**128**) was converted into the allose-containing trisaccharide (**127**) via inversion of the equatorial alcohol (OH-3^Glc^) to an axial configuration. In contrast, sodium decatungstate (NaDT)-promoted conditions resulted in selective inversion of the axial alcohol (OH-4^Gal^) to the equatorial orientation found in *d*-theanderose (**129**). This work showcases the utility and precision of tailored isomerization reactions for the interconversion of highly oxidized complex glycans without using any protecting groups.

### Aspirational Goals for Stereochemical Editing Strategies

Stereochemical editing approaches are gaining traction in natural product total synthesis, with recent developments moving beyond classical α-to-carbonyl examples.^166,167^ These strategies represent a growing toolkit for stereochemical editing; however, their application in natural product total synthesis remains limited. Photocatalytic strategies have enabled site-selective epimerization and deracemization of sugars, cyclic diols, as well as cyclic α-aryl ketones.^165,168–171^ Various groups also disclosed complementary radical approaches that enable stereochemical editing of less activated sites.^172,173^ Stereochemical editing via C–C bond cleavage and reformation has also been achieved using photochemical methods.^174,175^ Notably, Schuppe and co-workers reported the epimerization of quaternary carbons in steroidal frameworks (such as C13 in **130**), enabling the enantioselective synthesis of habiterpenol (**132**) and dasyscyphin A (Scheme 10F).^176^ In these transformations, the carbon skeleton is temporarily cleaved, enabling a sampling of conformations and selective editing of a stereocenter while preserving connectivity. As photochemical, electrochemical, transition metal catalyzed, and enzymatic methods continue to evolve, stereochemical editing will assume an increasingly important role in the field of skeletal editing and its applications to total synthesis.

The long-term vision of stereochemical editing is to be able to rewrite stereochemical information at will, without altering connectivity or resorting to lengthy detours. Further development in this area would grant chemists additional versatility in natural product synthesis, late-stage diversification, and lead optimization. While single stereocenter editing represents a major advance, the broader aspiration is to modify multiple stereocenters within complex frameworks, including contra-thermodynamic epimerization beyond α-to-carbonyl positions. Particularly challenging, yet highly desirable, is the editing of tetrasubstituted carbons across a broad range of substrates. Achieving such transformations will likely require new methods for non-C–H bond-based stereochemical editing, potentially exploiting alternative bond homolysis events to enable stereochemical epimerization. Ultimately, the ability to invert fully substituted stereocenters would mark a fundamental shift in synthetic strategy, particularly if extended beyond central chirality to axial, planar, and other stereogenic elements such as biaryl atropisomers.

## Conclusions

Since the very beginning of dedicated efforts to construct natural products, chemists have attempted to mimic Mother Nature, driving innovation in synthetic chemistry with the potential to exceed what is possible in nature. Recently, single-atom skeletal edits have been invoked in the biosynthetic pathways for a range of natural product scaffolds, through both enzymatic and nonenzymatic pathways.^177^ These naturally occurring single-atom skeletal manipulations lay the groundwork for organic chemists to incorporate analogous transformations into their synthetic designs.

In the preparation of this Perspective, we observed several trends among the types of skeletal edits that enabled strategic and efficient total synthesis. First, skeletal edits applied to readily available or easily synthesized molecular scaffolds allow for the rapid construction of more complex and often more synthetically challenging skeletons. For example, methods that enabled stereocontrolled formation of contiguous stereocenters, often through single-atom deletions and ring contractions, provide swift access to highly congested polycyclic cores. Second, many of the skeletal editing methods that prove useful in total synthesis also enable downstream modification through the introduction of a functional handle, often for cross-coupling or olefination. These trends are most common in single-atom insertions and ring contractions, but novel transmutation methods could provide access to equally versatile products. Third, the ability to interconvert between different ring systems (both in size and atom identity) is a powerful tool for circumventing chemical incompatibility. These skeletal edits allow chemists to perform early stage manipulations on precursor molecular scaffolds before accessing the desired ring system, which would otherwise lack the corresponding reactivity and not undergo the requisite transformations. Lastly, although this Perspective is focused on the applications of the concept of skeletal editing in total synthesis, we acknowledge that the continued development of robust and selective methodologies is also guided by unmet needs in industry, such as medicinal and process chemistry efforts.^178^ While single-atom skeletal edits are valuable strategies for modifying molecular frameworks, their adoption in industry remains limited. This is largely because practical and effective methods are typically restricted to a narrow set of functional groups and can require expensive reagents or harsh conditions that are not readily performed on industrial scale. We hope that the lessons learned from the existing literature can serve as guidelines for the development of other impactful single-atom skeletal editing methods. Ultimately, the field of skeletal editing should aspire to intuitively and efficiently delete, insert, or mutate the atoms of a molecular scaffold with precision and purpose.

In this Perspective, we have reframed both classical and modern examples of skeletal editing through a unified lens, highlighting a mode of synthetic planning in which skeletal editing is strategically integrated into the design from the outset, rather than applied retroactively. Guided by Corey’s retrosynthetic logic,^179^ chemists are now “encoding” precursors–intermediates that contain latent reactivity that can be selectively triggered to access products with desired functionalities. In the context of skeletal editing, this “retrosynthetic encoding” allows chemists to realize a programmed, atom-level transformation of the core framework, often at a late stage of a synthetic sequence. This concept aligns with a broader shift toward programmable synthesis, wherein latent reactivity is embedded in the structure and revealed on demand by specific chemical inputs. The emerging capabilities of artificial intelligence and retrosynthesis algorithms suggest that computational systems are beginning to recognize a key insight of modern synthetic planning: that molecular skeletons are not static and can be treated as reprogrammable frameworks.^45^ Skeletal editing strategies complement traditional approaches to total synthesis, advancing a mindset in which editable nodes, or sites where a single atom can be inserted, deleted, or transmuted, are intentionally encoded into synthetic routes.

## ∥

Department of Biochemistry, The University of Texas Southwestern Medical Center, Dallas, Texas 75390, United States

## Abbreviations

*m*-CPBA: meta-chloroperbenzoic acid
RAR: radical anion rearrangement
TMSCN: trimethylsilyl cyanide
IBX: 2-iodoxybenzoic acid
ODI: oxidative dearomatization induced
MHAT: metal-catalyzed hydrogen atom transfer
ANRORC: addition of a nucleophile, ring opening, and ring closure
HAA: hydrogen atom abstraction
NaDT: sodium decatungstate
AvmO1: alchivemycin monooxygenase enzyme O1 variant
